# The effect of postoperative chemotherapy on survival outcomes and a nomogram for predicting overall survival in chondroblastic osteosarcoma

**DOI:** 10.1038/s41598-025-31032-y

**Published:** 2025-12-29

**Authors:** Ruicong Li, Huiying Wang, Weipeng Zheng, Ning Wang, Zekai Ma, Shifeng Wen

**Affiliations:** 1 Department of Orthopedics, Guangzhou First People’s Hospital, Guangzhou Medical University, Guangzhou, Guangdong People’s Republic of China; 2 Guangzhou First People’s Hospital, Guangzhou Medical University, Guangzhou, Guangdong People’s Republic of China; 3 Guangzhou United Family Hospital, Guangzhou, Guangdong People’s Republic of China; 4 Guangzhou First People’s Hospital, Guangzhou, Guangdong People’s Republic of China; 5 Department of Orthopedics, Guangzhou First People’s Hospital, Guangzhou Medical University, Guangzhou, Guangdong People’s Republic of China; 6 Department of Orthopaedic Surgery, Guangzhou First People’s Hospital, the Second Affiliated Hospital, School of Medicine, South China University of Technology, Guangzhou, Guangdong, People’s Republic of China

**Keywords:** Chondroblastic osteosarcoma, Postoperative chemotherapy, Prognostic nomogram, Overall survival, Cancer, Medical research, Oncology

## Abstract

The analysis of chondroblastic osteosarcoma (CBO) was conducted using data from the Surveillance, Epidemiology, and End Results (SEER) database (2000–2019).​ A cohort of 317 surgically treated CBO patients was analyzed, with 261 (82.3%) receiving adjuvant chemotherapy and 56 (17.7%) classified as no/unknown chemotherapy recipients. The median diagnosis year of CBO patients was 2005 and the median follow-up time was 5.25 years (63 months).​​ Kaplan-Meier analysis demonstrated that chemotherapy significantly improved overall survival (OS) (*P* = 0.011), a finding corroborated after propensity score matching (*P* = 0.036). Specifically, the 5-year, 10-year, and 15-year overall survival(OS) of patients who received chemotherapy were 52.11%, 35.25%, and 25.29%, respectively. In contrast, the corresponding survival rates of patients who did not receive chemotherapy or whose chemotherapy status was unknown were 48.21%, 39.29%, and 25.00%, respectively. Subgroup analyses revealed enhanced chemotherapy efficacy in males (HR = 0.48, *P* = 0.01), high grade tumors (HR = 0.54, *P* = 0.015), and high income patients (HR = 0.35, *P* = 0.001), while ​stage stratified analysis showed that the overall survival benefit of chemotherapy was limited to patients with stage I–II disease (P for interaction = 0.008). Further interaction analysis revealed that the survival benefit of chemotherapy in patients with stage III–IV disease was significantly attenuated compared to those with stage I–II disease (HR = 0.06, *P* = 0.049), indicating a pronounced stage dependent effect. Multivariate Cox regression identified age, tumour stage and radiotherapy as independent prognostic factors. The constructed nomogram integrating these factors with marital status and chemotherapy achieved C-indices of 0.674 (training set) and 0.691 (validation set). Notably, it exhibited strong discriminative ability with AUC values of 0.672, 0.658, and 0.657 for predicting 5-, 10-, and 15-year OS in the training set, and even higher values of 0.741, 0.712, and 0.745 in the validation set. This tool provides individualized 5-, 10-, and 15-year OS predictions, addressing critical gaps in CBO specific prognostic assessment and supporting personalized therapeutic decision making.

**Supplementary Information:**

The online version contains supplementary material available at 10.1038/s41598-025-31032-y.

## Introduction

Chondroblastic osteosarcoma (CBO) is a rare, clinically and epigenetically distinct variant of chondroblastic tumors^1^. It is characterized by its unique histological features, including chondroblastic -like cells and chondroid matrix formation, and typically arises in the epiphyses of long bones in adolescents and young adults^1,2^. Chondroblastic osteosarcoma accounts for approximately 20–25% of all osteosarcoma cases, making it one of the most common histological subtypes^3^. It predominantly affects adolescents and young adults aged 10–25 years, with a slight male predominance, and most commonly arises in the metaphyseal regions of long bones, particularly the distal femur, proximal tibia, and proximal humerus^4,5^. Although the overall incidence of CBO is low, its relative proportion within osteosarcoma highlights its clinical importance. Furthermore, its potential for local aggressiveness and metastasis poses significant clinical challenges, necessitating a better understanding of its natural history and optimal treatment strategies. Osteosarcoma, the most common primary malignant bone tumor overall, has seen significant improvements in survival over recent decades, largely attributed to the advent of multi-agent neoadjuvant and adjuvant chemotherapy^6,7^. However, the specific impact of chemotherapy on the survival outcomes of patients with the chondroblastic subtype remains poorly defined and is a subject of ongoing debate, primarily due to the extreme rarity of the disease which has precluded large-scale randomized studies.

The etiology of CBO is not fully elucidated like other osteosarcomas. However, it is associated with factors such as rapid bone growth during adolescence, pre-existing Paget’s disease of bone, and genetic predispositions including mutations in the RB1 and TP53 genes^8,9^. The primary treatment for localized osteosarcoma has evolved to include wide surgical resection combined with perioperative chemotherapy, which has dramatically increased the 5-year survival rate from less than 20% with surgery alone to approximately 60–70%^6,10,11^. For conventional osteosarcoma, the role of chemotherapy is well-established; it aims to eradicate micrometastatic disease, reduce tumor volume to facilitate limb-salvage surgery and improve overall survival. Nevertheless, the biological behavior and chemosensitivity of the chondroblastic variant are believed to differ from the more common osteoblastic subtype^12^. Some small series and case reports have suggested that CBO may have a more indolent course and potentially a better prognosis^1,13^. Nevertheless, emerging evidence suggests that this tumor subtype may also demonstrate a propensity for delayed disease recurrence and metastatic dissemination, with pulmonary metastases representing the predominant metastatic pattern and primary contributor to mortality^14^.

Particularly those with pulmonary metastases, patients who develop metastatic disease have a poor prognosis and their long-term survival rates decrease markedly^15^. Bone is a prevalent metastatic site for many sarcomas. These metastases worsen the clinical picture, leading to skeletal-related events (SREs) like pathological fractures, spinal cord compression, and intense pain that drastically reduce the quality of life^16,17^. Accurate prognosis prediction is crucial for tailoring aggressive treatment for high-risk patients while potentially sparing low-risk patients from the severe toxicity of unnecessary chemotherapy. Current staging protocols, including the widely adopted AJCC TNM system, offer valuable but limited prognostic guidance for this rare malignancy. Their primary shortcoming lies in the lack of integration of essential clinicopathological and therapeutic factors, thereby failing to support finely granular, individualized outcome predictions.

In recent years, nomograms have emerged as invaluable and reliable statistical tools in oncology for predicting individual cancer prognosis^18^. They integrate multiple independent prognostic factors to generate a personalized numerical probability of clinical events, such as overall survival (OS) or cancer specific survival (CSS). Such predictive tools demonstrate enhanced prognostic precision over conventional staging methods. Their application across multiple cancer types facilitates improved risk stratification, enhances patient consultation and informs the development of tailored management strategies. Key factors commonly incorporated into such models include demographic variables (e.g., age), tumor characteristics (e.g., size, grade, primary site), disease extent (TNM stage, presence of metastasis), and treatment modalities received (e.g., surgery, chemotherapy, radiotherapy)^19,20^. For osteosarcoma, several nomograms have been established^18,21^. But none specifically address the chondroblastic subtype due to its rarity.

Given the critical need to clarify the effect of chemotherapy on survival in this unique population and the absence of a tailored prognostic tool, this study aims to investigate the impact of chemotherapy on overall survival outcomes in patients with chondroblastic osteosarcoma. Furthermore, we seek to develop and validate a comprehensive nomogram for predicting overall survival in these patients. Utilizing a large, population-based national cancer registry—the Surveillance, Epidemiology, and End Results (SEER) database—we identified a sufficiently sized cohort of patients diagnosed with chondroblastic osteosarcoma. By integrating key clinicopathological and treatment variables, including chemotherapy status, this research aims to provide a practical tool for clinicians to improve prognostic assessment and inform personalized therapeutic strategies for patients with this rare and challenging disease.

## Materials and methods

### Patient acquisition and data processing

Data were obtained from the SEER database (2000–2019), comprising de-identified public records from 17 registries. These 17 registries cover approximately 34.6% of the U.S. population and span multiple geographic regions, ensuring the geographic representativeness of the study population. Variable extraction was performed using SEER^*^Stat software (version 9.0.41). This study adhered to the Declaration of Helsinki. Ethical review was waived per SEER protocol as data were pre-authorized for research use, requiring no additional patient consent.

Although the SEER database does not provide information on the specific hospitals or treatment centers where patients received care, all included patients were treated at healthcare facilities within the SEER-covered registry regions. These facilities include community hospitals, general hospitals, academic medical centers, and specialized cancer centers, reflecting the diversity of treatment settings in routine U.S. clinical practice. In addition, SEER provides the Rural-Urban Continuum Code (RUCC), which allows for the classification of patients’ residential areas by urban or rural attributes, although it does not specify the type of treatment institution.

**Fig. 1:**
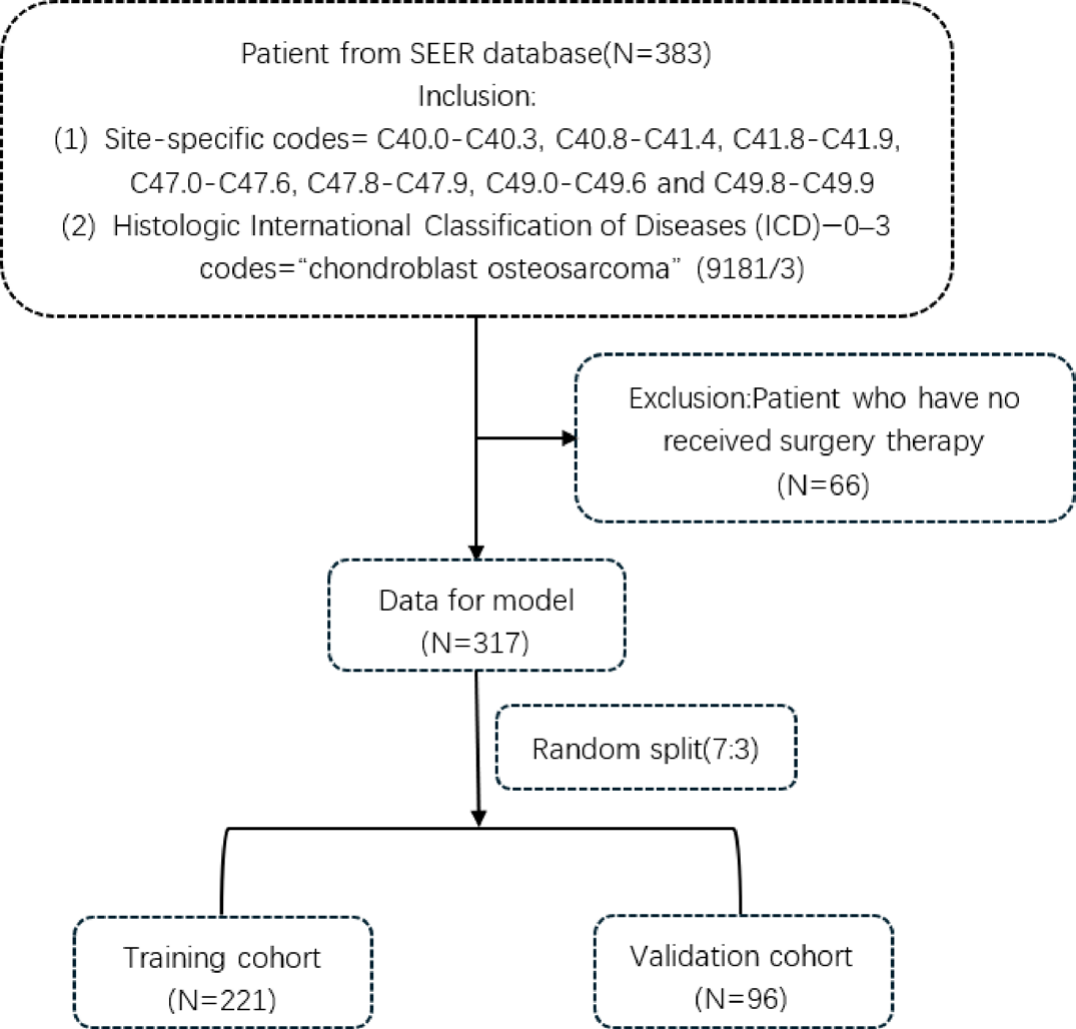
Flow chart detailing the selection of the patients in this study.

Primary tumors originating in soft tissue, bone, and joints were identified using site-specific codes (C40.0–C40.3, C40.8–C41.4, C41.8–C41.9, C47.0–C47.6, C47.8–C47.9, C49.0–C49.6, C49.8–C49.9). Histologic subtypes were further selected according to ICD-O-3 codes: chondroblastic osteosarcoma (9181/3). The extracted variables included age, sex, race, median household income, marital status at diagnosis, tumor grade, stage (based on the 6th edition AJCC staging system), treatment records (surgery, radiotherapy, chemotherapy), survival months, vital status, and Rural-Urban Continuum Code. All variables are based on clinical relevance and previous literature. Overall survival (OS) served as the primary outcome of this study. Initial screening identified 383 eligible patients diagnosed with the condition. After excluding 66 patients who did not undergo surgical treatment, a total of 317 patients were included in the final study cohort. The research flowchart is shown in Fig. 1.

### Statistical analysis and nomogram development

Descriptive statistics were initially used to compare baseline characteristics of individuals based on their receipt of adjuvant chemotherapy. The Chi-square test was employed for comparing categorical variables, while the independent t-test was used for continuous variables. Kaplan-Meier survival estimates were applied to evaluate overall survival outcomes according to adjuvant chemotherapy status. Additionally, propensity score matching was implemented between patients who received adjuvant radiotherapy and those who did not (i.e., the no/unknown category). Matching was performed based on age, sex, race, median household income, marital status at diagnosis, tumor grade, stage, radiotherapy status, and Rural-Urban Continuum.

**Fig. 2:**
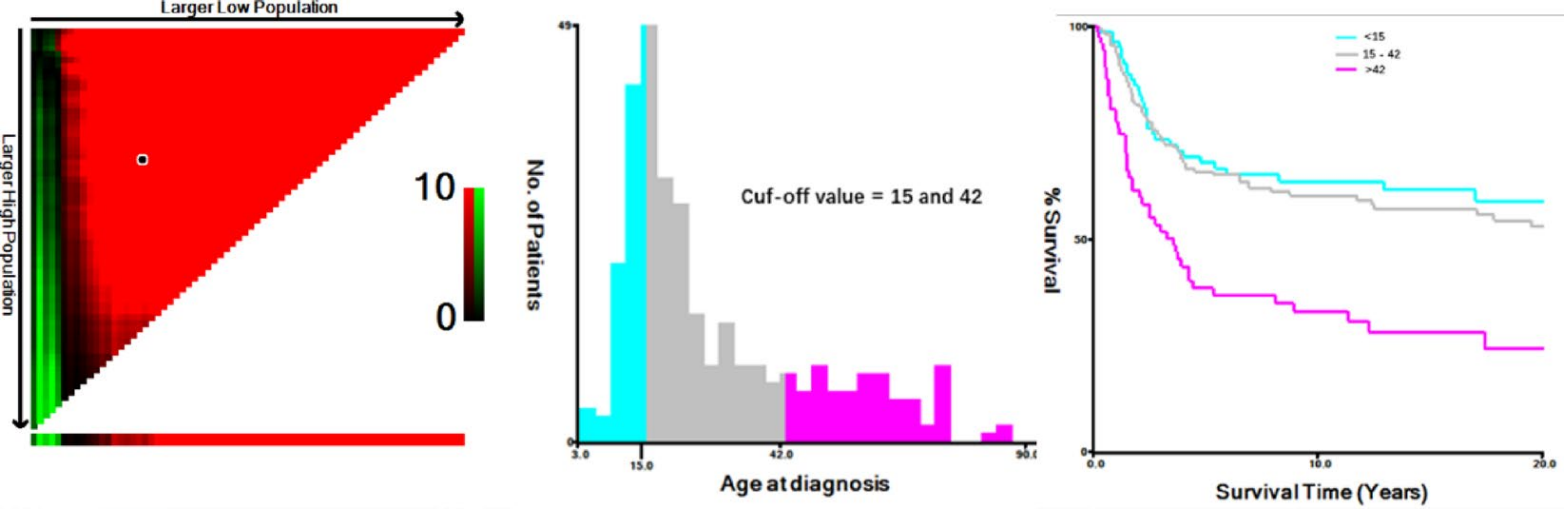
Determination of optimal age cut-off values using X-tile software.

For the construction and validation of the nomogram, subjects were randomly divided into training and validation cohorts in a 7:3 ratio. The Chi-square test was utilized for analyzing percentage variance in count data. Optimal cut-off values for age were determined using X-tile software, with results presented in Fig. 2. Univariate and multivariate Cox regression analyses were subsequently conducted to identify factors associated with inferior overall survival.

Based on these identified prognostic factors, a nomogram was developed to predict 5-, 10-, and 15-year OS in patients with chondroblastic osteosarcoma. The prognostic nomogram underwent both internal and external validation using the training and validation cohorts, respectively. Harrell’s concordance index (C-index) and the area under the receiver operating characteristic curve (AUC) served as key metrics for evaluating the performance of the nomogram. All statistical analyses were performed using R software (version 4.5.0). The p-value < 0.05 was considered statistically significant.

The optimal cutoff values were identified for age at diagnosis as 15 and 42 years based on overall survival and analysis using X-tile software.

## Results

### Patient characteristics

A total of 317 patients were included in this study. These included 261 (82.3%) patients who received adjuvant chemotherapy and 56 (17.7%) who did not receive chemotherapy or had unknown treatment status. Intergroup comparisons revealed that patients receiving adjuvant chemotherapy were significantly younger (*P* < 0.001), with a higher proportion of males and unmarried individuals (*P* = 0.024; *P* < 0.001). Significant differences were also observed in tumor stage and grade distribution between the two groups (*P* = 0.002; *P* = 0.006). However, no statistically significant differences were found in race (*P* = 0.500), radiotherapy application (*P* = 0.415), income level (*P* = 0.211), or rural-urban continuum (*P* = 0.061) (Table 1). These findings suggest that the selection of adjuvant chemotherapy is closely associated with patient age, sex, marital status, and tumor malignancy.

**Table 1:** Baseline characteristics of included patients in the current study (317 patients).

Variable	Those who received adjuvant chemotherapy therapy (*N* = 261)	No/unknown chemotherapy therapy (*N* = 56)	Overall (*N* = 317)	*P*-value
Age, years (mean ± SD)	25.72 ± 15.93	44.43 ± 23.52	29.03 ± 18,87	< 0.001
Sex				0.024
Male	162 (62.1%)	25 (44.6%)	187 (59.0%)	
Female	99 (37.9%)	31 (55.4%)	130 (41.0%)	
Race				0.500
White	188 (72.0%)	43 (76.8%)	231 (72.9%)	
Black	36 (13.8%)	9 (16.1%)	45 (14.2%)	
Others	33 (12.6%)	4 (7.1%)	37 (11.7%)	
Unknown	4 (1.5%)	0 (0%)	4 (1.3%)	
Marital status				< 0.001
DSW	11 (4.2%)	6 (10.7%)	17 (5.4%)	
Married	62 (23.8%)	25 (44.6%)	87 (27.4%)	
Single	185 (70.9%)	22 (39.3%)	207 (65.3%)	
Unknown	3 (1.1%)	3 (5.4%)	6 (1.9%)	
Radiotherapy				0.415
Yes	17 (6.5%)	6 (10.7%)	23 (7.3%)	
No/Unknown	244 (93.5%)	50 (89.3%)	294 (92.7%)	
Stage				0.002
I	4 (1.5%)	6 (10.7%)	10 (3.2%)	
II	50 (19.2%)	5 (8.9%)	55 (17.4%)	
III	3 (1.1%)	0 (0%)	3 (0.9%)	
IV	12 (4.6%)	1 (1.8%)	13 (4.1%)	
Unknow	192 (73.6%)	44 (78.6%)	236 (74.4%)	
Grade				0.006
I	3 (1.1%)	3 (5.4%)	6 (1.9%)	
II	7 (2.7%)	6 (10.7%)	13 (4.1%)	
III	62 (23.8%)	16 (28.6%)	78 (24.6%)	
IV	82 (31.4%)	11 (19.6%)	93 (29.3%)	
Unknow	107 (41.0%)	20 (35.7%)	127 (40.1%)	
Income				0.211
< $75,000	52 (19.9%)	12 (21.4%)	64 (20.2%)	
$75,000–90,000	83 (31.8%)	16 (28.6%)	99 (31.2%)	
≥ $90,000	96 (36.8%)	16 (28.6%)	112 (35.3%)	
Unknown	30 (11.5%)	12 (21.4%)	42 (13.2%)	
Rural-urban continuum				0.061
Metropolitan areas	202 (77.4%)	35 (62.5%)	237 (74.8%)	
Nonmetropolitan counties	28 (10.7%)	9 (16.1%)	37 (11.7%)	
Unknown	31 (11.9%)	12 (21.4%)	43 (13.6%)	

DSW, divorced&Separated&Widowed.

**Fig. 3:**
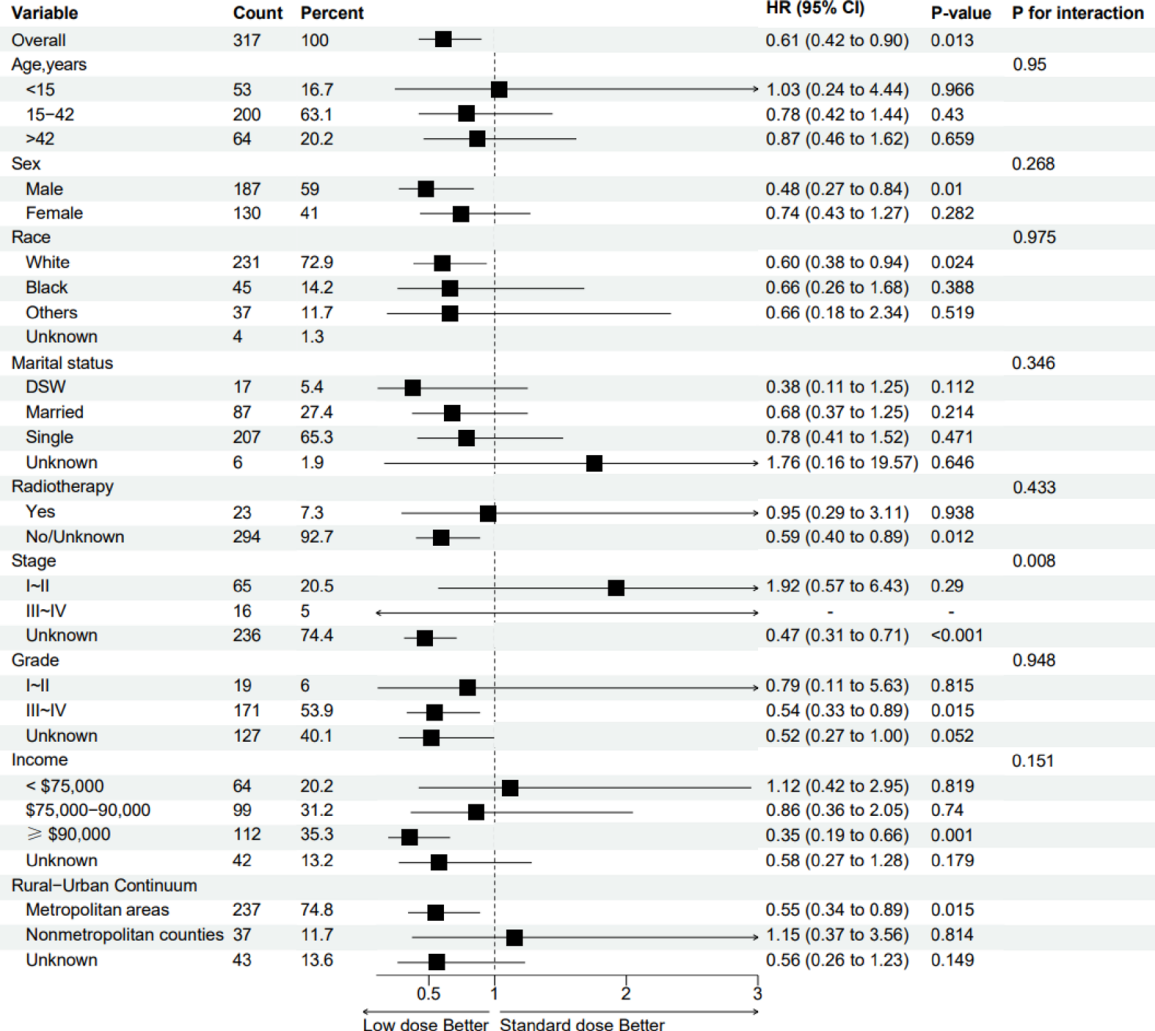
Forest plot of postoperative chemotherapy effects with subgroup analysis in chondroblastic osteosarcoma. DSW, divorced&Separated&Widowed; CI, confidence interval; HR, hazard ratio.

Figure 3 presents a forest plot illustrating the primary effect of postoperative chemotherapy and subgroup heterogeneity in patients with chondroblastic osteosarcoma. Subgroup analyses demonstrated differential efficacy of chemotherapy, with significant survival benefits observed in males (HR = 0.48, *P* = 0.01), patients with high grade tumors (HR = 0.54, *P* = 0.015), and high income subgroups (HR = 0.35, *P* = 0.001). Notably, a significant interaction was identified between tumor stage and chemotherapy efficacy (P for interaction = 0.008). No significant heterogeneity in treatment effect was observed across other subgroups, including age, race, marital status, and radiotherapy (P for interaction > 0.05).

### Outcome of overall survival according to postoperative chemotherapy

**Fig. 4:**
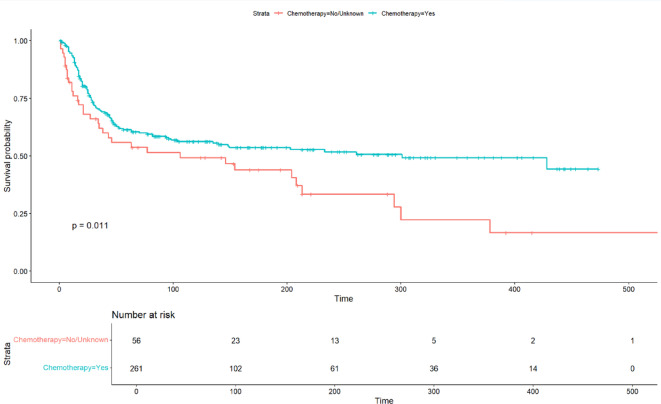
Overall survival according to the use of postoperative chemotherapy.

Using the Kaplan-Meier method, individuals who received postoperative chemotherapy demonstrated significantly improved overall survival (*P* = 0.011; Fig. 4).

### Survival outcomes in the post-propensity cohort

**Fig. 5:**
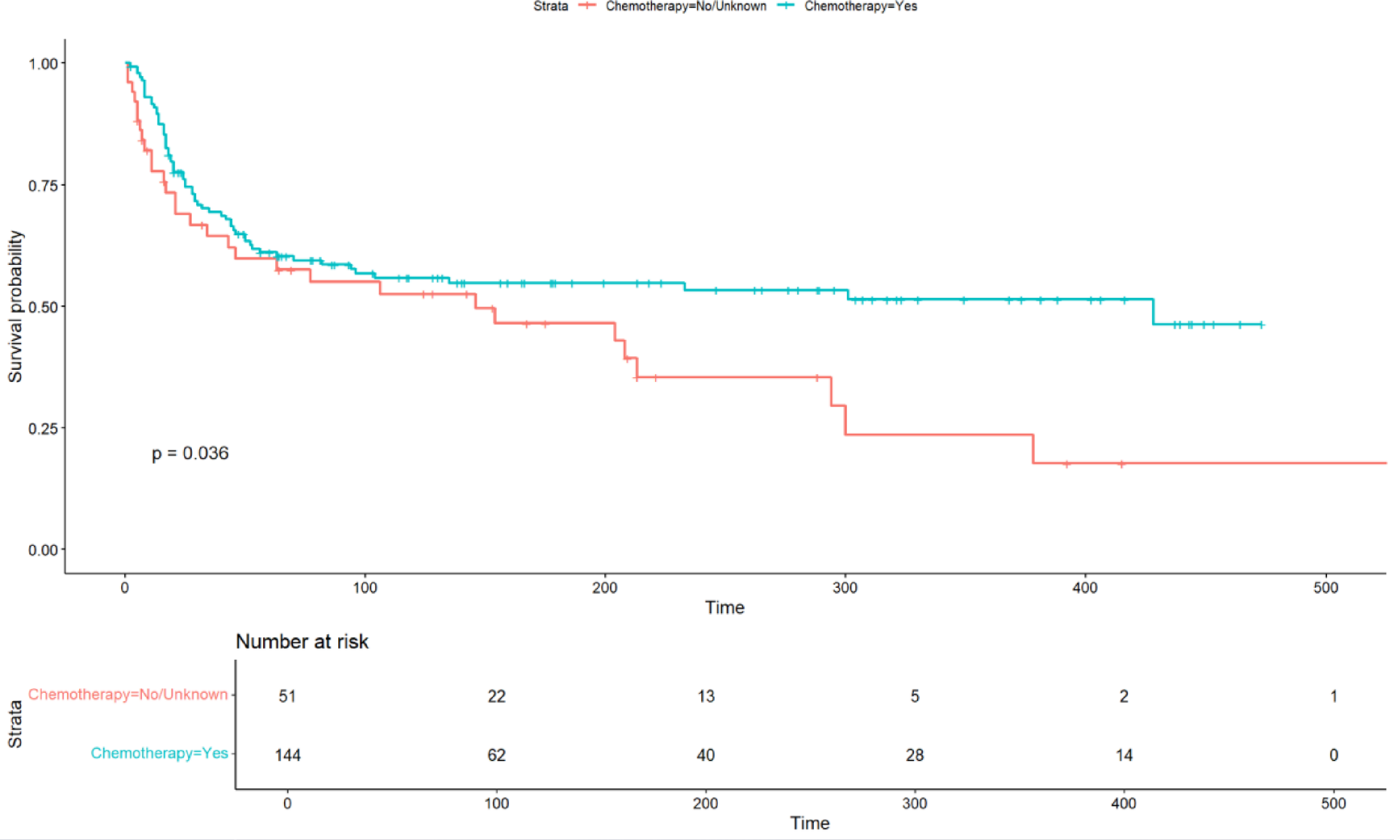
Overall survival according to the use of postoperative chemotherapy (post-propensity cohort).

Following propensity score matching using a 4:1 ratio and a caliper width set at 0.7 times the standard deviation of the propensity score, a total of 144 patients who received postoperative chemotherapy were successfully matched with 51 patients in the no/unknown treatment category (Table 2). Among the original 56 patients in the no/unknown group, not all were matched due to limitations in the number of available matches or propensity score distances exceeding the predefined caliper. As a result, 51 patients from this group were included in the final matched cohort, while the remaining 5 were not matched and thus excluded (Supplementary Fig. 1). Patients receiving postoperative chemotherapy demonstrated significantly improved overall survival in the post-matching cohort (*P* = 0.036; Fig. 5). The distribution of propensity scores before and after matching is detailed in the histogram presented in Supplementary Fig. 2.

**Table 2:** Baseline characteristics of patients with chondroblastic Osteosarcoma after propensity score matching (195 patients).

Variable	Those who received adjuvant Chemotherapy therapy (144 patients)	No/unknown Chemotherapy therapy (51 patients)	Overall (195 patients)	*P*-value
Age, years (mean ± SD)	32.16 ± 17.84	41.90 ± 22.89	34.71 ± 19.70	0.057
Sex				0.436
Male	76 (52.8%)	23 (45.1%)	99 (50.8%)	
Female	68 (47.2%)	28 (54.9%)	96 (49.2%)	
Race				0.996
White	111 (77.1%)	39 (76.5%)	150 (76.9%)	
Black	22 (15.3%)	8 (15.7%)	30 (15.4%)	
Others	11 (7.6%)	4 (7.8%)	15 (7.7%)	
Marital status				0.573
DSW	10 (6.9%)	5 (9.8%)	15 (7.7%)	
Married	54 (37.5%)	22 (43.1%)	76 (39.0%)	
Single	77 (53.5%)	22 (43.1%)	99 (50.8%)	
Unknown	3 (2.1%)	2 (3.9%)	5 (2.6%)	
Radiotherapy				0.885
Yes	14 (9.7%)	6 (11.8%)	20 (10.3%)	
No/Unknown	130 (90.3%)	45 (88.2%)	175 (89.7%)	
Stage				0.928
I–II	26 (18.1%)	10 (19.6%)	36 (18.5%)	
III–IV	4 (2.8%)	1 (2.0%)	5 (2.6%)	
Unknown	114 (79.2%)	40 (78.4%)	154 (79.0%)	
Grade				0.520
I–II	10 (6.9%)	6 (11.8%)	16 (8.2%)	
III–IV	73 (50.7%)	26 (51.0%)	99 (50.8%)	
Unknown	61 (42.4%)	19 (37.3%)	80 (41.0%)	
Income				0.886
< $75,000	38 (26.4%)	11 (21.6%)	49 (25.1%)	
$75,000–90,000	41 (28.5%)	15 (29.4%)	56 (28.7%)	
≥ $90,000	36 (25.0%)	15 (29.4%)	51 (26.2%)	
Unknown	29 (20.1%)	10 (19.6%)	39 (20.0%)	
Rural–urban continuum				0.981
Metropolitan areas	94 (65.3%)	33 (64.7%)	127 (65.1%)	
Nonmetropolitan counties	21 (14.6%)	8 (15.7%)	29 (14.9%)	
Unknown	29 (20.1%)	10 (19.6%)	39 (20.0%)	

DSW, divorced&Separated&Widowed.

###  Baseline characteristics of the training and validation cohorts

Furthermore, the 317 patients were randomly allocated into a training cohort (*n* = 221) and a validation cohort (*n* = 96) in a 7:3 ratio. No significant differences were identified between the training and validation cohorts regarding patient age, sex, race, marital status, tumor stage, grade, income, rural-urban distribution, utilization of chemotherapy, or application of radiotherapy, confirming the comparability between the two groups(Table 3). The training cohort was allocated for the construction and internal validation of the nomogram, while the validation cohort was designated for external validation of the nomogram.

**Table 3:** Population baseline characteristics of training set and validation set.

Variable	Training (*N* = 221)	Validation (*N* = 96)	Overall (*N* = 317)	χ²	*P*-value
Age, years				0.907	0.635
< 15	46 (20.8%)	24 (25.0%)	70 (22.1%)		
15–42	121 (54.8%)	52 (54.2%)	173 (54.6%)		
> 42	54 (24.4%)	20 (20.8%)	74 (23.3%)		
Sex				1.054	0.305
Male	135 (61.1%)	52 (54.2%)	187 (59.0%)		
Female	86 (38.9%)	44 (45.8%)	130 (41.0%)		
Race				1.833	0.608
White	161 (72.9%)	70 (72.9%)	231 (72.9%)		
Black	31 (14.0%)	14 (14.6%)	45 (14.2%)		
Others	25 (11.3%)	12 (12.5%)	37 (11.7%)		
Unknown	4 (1.8%)	0 (0%)	4 (1.3%)		
Marital status				0.754	0.86
DSW	12 (5.4%)	5 (5.2%)	17 (5.4%)		
Married	62 (28.1%)	25 (26.0%)	87 (27.4%)		
Single	142 (64.3%)	65 (67.7%)	207 (65.3%)		
Unknown	5 (2.3%)	1 (1.0%)	6 (1.9%)		
Chemotherapy				0.219	0.64
Yes	41 (18.6%)	15 (15.6%)	261 (82.3%)		
No/Unknown	180 (81.4%)	81 (84.4%)	56 (17.7%)		
Radiotherapy				2.666	0.103
Yes	20 (9.0%)	3 (3.1%)	23 (7.3%)		
No/Unknown	201 (91.0%)	93 (96.9%)	294 (92.7%)		
Stage				5.429	0.066
I–II	51 (23.1%)	14 (14.6%)	65 (20.5%)		
III–IV	8 (3.6%)	8 (8.3%)	16 (5.0%)		
Unknown	162 (73.3%)	74 (77.1%)	236 (74.4%)		
Grade				0.043	0.979
I–II	13 (5.9%)	6 (6.3%)	19 (6.0%)		
III–IV	120 (54.3%)	51 (53.1%)	171 (53.9%)		
Unknown	88 (39.8%)	39 (40.6%)	127 (40.1%)		
Income				0.242	0.971
< $75,000	44 (19.9%)	20 (20.8%)	64 (20.2%)		
$75,000–90,000	68 (30.8%)	31 (32.3%)	99 (31.2%)		
≥ $90,000	80 (36.2%)	32 (33.3%)	112 (35.3%)		
Unknown	29 (13.1%)	13 (13.5%)	42 (13.2%)		
Rural-urban continuum				1.52	0.468
Metropolitan areas	163 (73.8%)	74 (77.1%)	237 (74.8%)		
Nonmetropolitan counties	29 (13.1%)	8 (8.3%)	37 (11.7%)		
Unknown	29 (13.1%)	14 (14.6%)	43 (13.6%)		

DSW, divorced&Separated&Widowed.

No significant differences were observed between the training and validation cohorts in terms of age, sex, race, marital status, tumor stage, grade, income, rural-urban distribution, use of chemotherapy or use of radiotherapy.

Univariate and multivariate Cox regression analysis for factors affecting overall survival of the training cohort.

Univariate Cox analysis based on the training cohort data revealed that four variables—age, marital status, tumor stage, and radiotherapy—were significantly associated with overall survival (OS) (*P* < 0.05). However, the remaining variables showed no statistical significance (Table 4). After adjusting for confounding factors through multivariate Cox analysis, age, marital status, radiotherapy, and tumor stage were identified as independent prognostic factors for OS (*P* < 0.05). Specifically, patients older than 42 years exhibited a significantly increased risk of mortality (HR = 2.867, 95% CI: 1.429–5.751, *P* = 0.013), whereas those who received radiotherapy or had unknown radiotherapy status demonstrated a reduced risk (HR = 0.449, 95% CI: 0.260–0.775, *P* = 0.016). Patients with advanced-stage (III–IV) tumors had the highest risk (HR = 4.667, 95% CI: 2.073–10.505, *P* = 0.002). Variables such as sex, race, and grade did not retain statistical significance after adjustment for confounders. Further interaction analysis revealed that the survival benefit of chemotherapy was significantly attenuated in patients with stage III–IV disease compared to those with stage I–II disease (HR = 0.06, 95% CI: 0.00-0.99, *P* = 0.049), indicating a pronounced stage dependent effect of chemotherapy on overall survival.​

**Table 4:** Univariate and multivariate Cox regression analysis for chondroblastic osteosarcoma (training cohort).

Covariates	Univariate Cox			Multivariate Cox		
	HR	95%CI	*P*-value	HR	95%CI	*P*-value
Age, years			< 0.001			
< 15	Reference			Reference		
15–42	1.348	0.831–2.187		1.284	0.767–2.148	0.425
> 42	2.888	1.727–4.828		2.867	1.429–5.751	0.013 *
Sex			0.946			
Male	Reference			Reference		
Female	1.014	0.723–1.421		0.759	0.515–1.117	0.241
Race			0.709			
White	Reference			Reference		
Black	1.343	0.869–2.074		1.087	0.677–1.746	0.772
Others	1.162	0.681–1.983		1.477	0.829–2.631	0.266
Unknown	0.759	0.145–3.981		0.797	0.146–4.360	0.826
Marital status			0.032			
DSW	Reference			Reference		
Married	0.492	0.264–0.916		0.591	0.286–1.221	0.233
Single	0.335	0.185–0.606		0.580	0.246–1.371	0.298
Unknown	0.355	0.098–1.286		0.347	0.084–1.432	0.219
Radiotherapy			0.049			
Yes	Reference			Reference		
No/Unknown	0.531	0.325–0.866		0.449	0.260–0.775	0.016 *
Stage			0.034			
I–II	Reference			Reference		
III–IV	3.624	1.758–7.474		4.667	2.073–10.505	0.002 **
Unknown	1.214	0.804–1.833		1.395	0.863–2.253	0.254
Grade			0.120			
I–II	Reference			Reference		
III–IV	2.615	0.988–6.921		2.442	0.888–6.716	0.146
Unknown	2.884	1.073–7.748		2.432	0.856–6.904	0.161
Income			0.216			
< $75,000	Reference			Reference		
$75,000–90,000	1.318	0.784–2.218		1.401	0.743–2.643	0.382
≥ $90,000	1.711	1.048–2.795		1.391	0.756–2.560	0.374
Unknown	1.790	1.007–3.180		1.698	0.844–3.419	0.213
Rural-urban continuum			0.215			
Metropolitan areas	Reference			Reference		
Nonmetropolitan counties	0.625	0.358–1.090		0.608	0.301–1.225	0.242
Unknown	1.209	0.771–1.895		–	–	–
Chemotherapy [no/unknown] × Stage [III–IV]	–	–	–	Reference		
Chemotherapy [yes] × Stage [III–IV]	–	–	–	0.06	0.00–0.99	0.049*

DSW, divorced&Separated&Widowed; CI, confidence interval; HR, hazard ratio.

### Constructing nomograms for OS

**Fig. 6:**
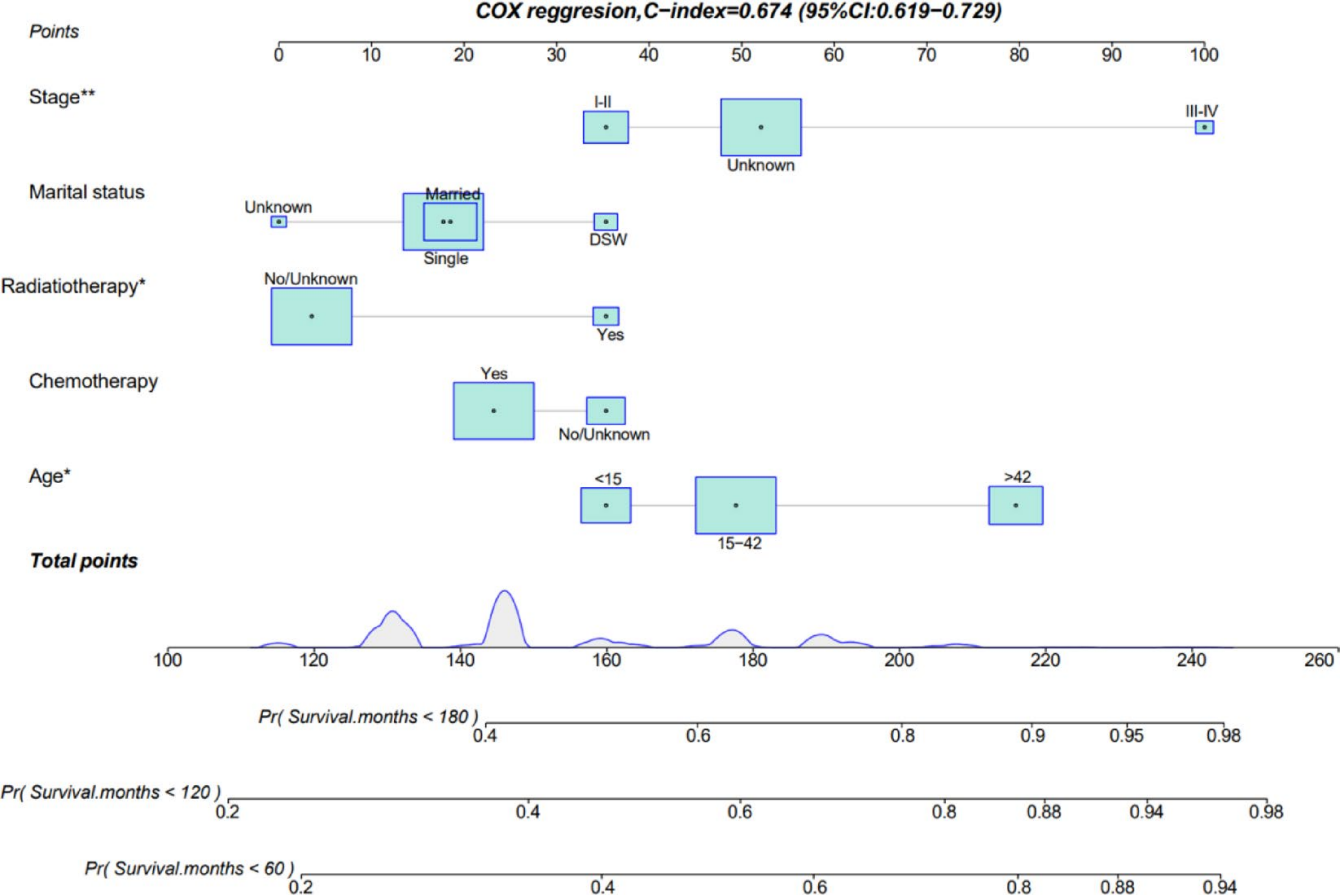
Prognostic nomogram for overall survival in chondroblastic osteosarcoma patients. DSW, divorced&Separated&Widowed; CI, confidence interval.

The nomogram derived from Cox regression analysis was constructed to estimate 5-, 10-, and 15-year overall survival (OS) in chondroblastic Osteosarcoma patients, integrating tumor stage, marital status, adjuvant radiotherapy, chemotherapy, and age at diagnosis (Fig. 6 and Supplementary Table 1). Tumor stage was identified as the most influential prognostic determinant, with radiotherapy status, marital status, and chemotherapy administration providing additional predictive value. For clinical application, the score corresponding to each variable is identified on its axis and summed to obtain a total point value. This aggregate score is then aligned with the survival probability scales to estimate OS rates at specified time intervals. The nomogram results for patient with Chondroblastic Osteosarcoma OS are shown in Fig. 6.

### Validation and calibration of the nomogram

**Fig. 7:**
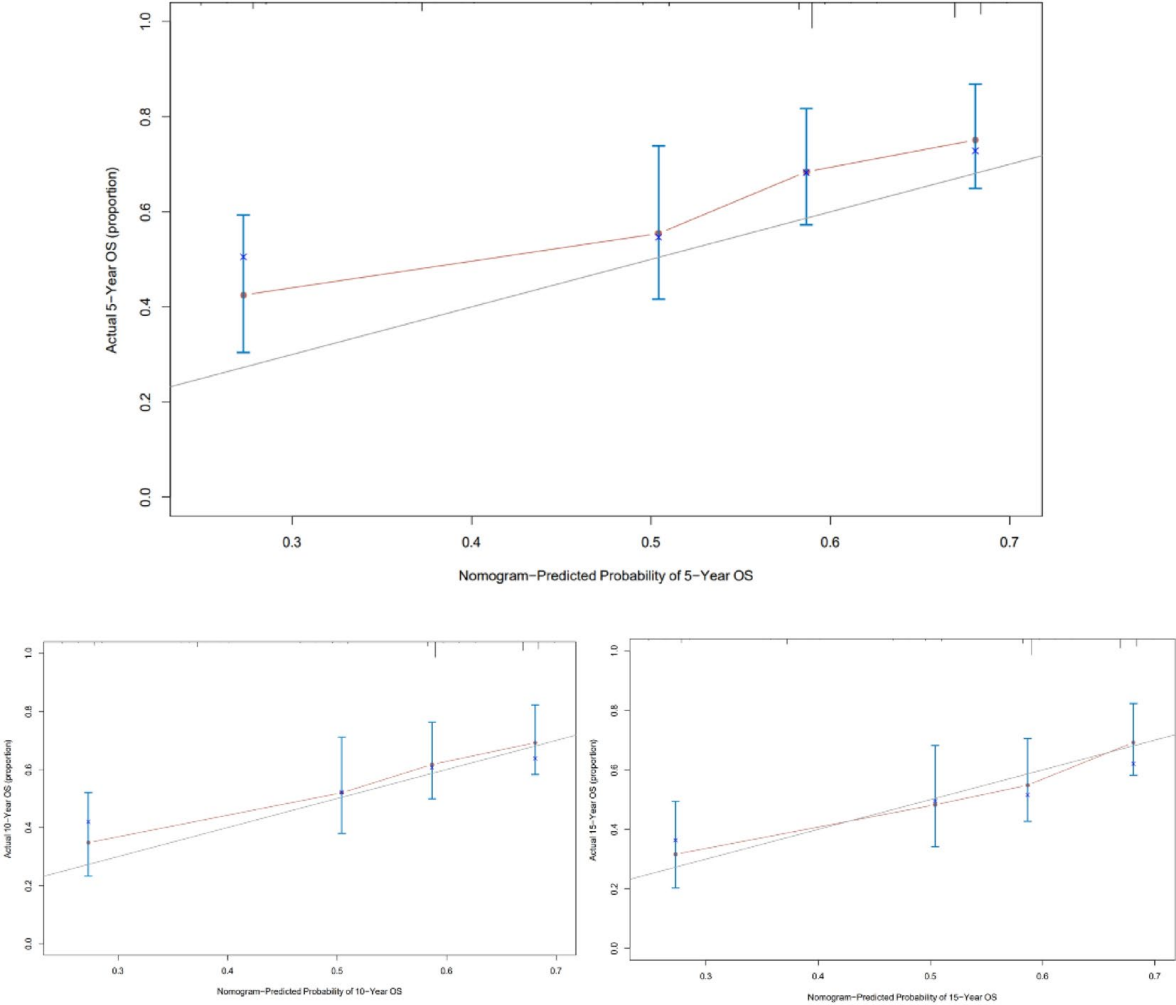
Internal calibration plots for 5-, 10-, and 15-year overall survival nomograms.

**Fig. 8:**
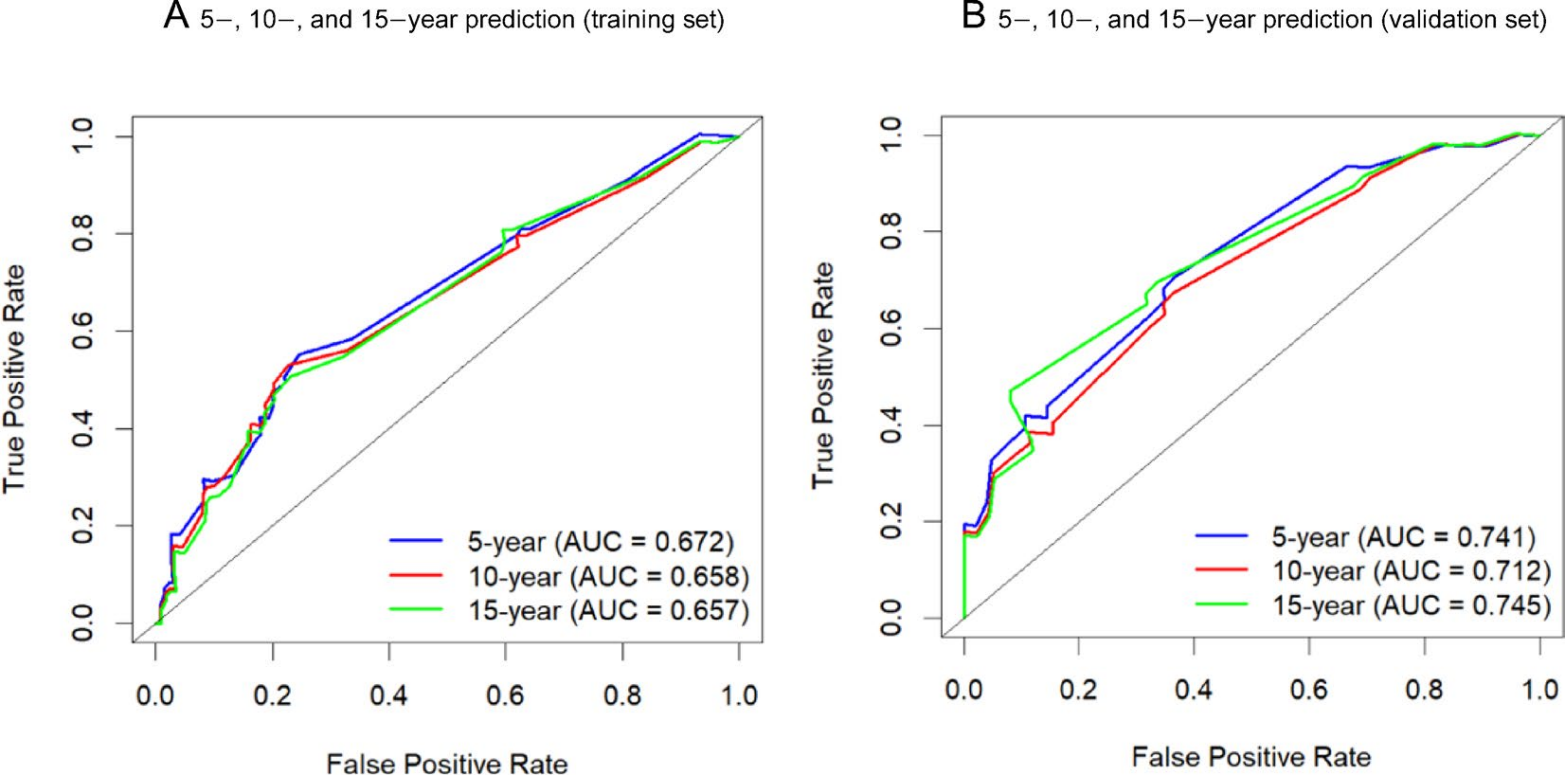
ROC curves of the nomogram for OS prediction: (**A**) training set; (**B**) validation set.

The predictive accuracy of the final prognostic nomogram model was assessed using the C-index. For the internal validation of the nomogram in the training cohort, the C-index for the OS nomogram was 0.674 (95% CI: 0.619–0.729). The externally validated C-index for the OS nomogram was 0.691 (95% CI: 0.607–0.775). Good agreement between the nomogram-predicted and actual survival rates was demonstrated in the calibration plot (Fig. 7). Moreover, ROC analysis indicated that the nomogram achieved area under the curve (AUC) values of 0.672, 0.658, and 0.657 for predicting 5-, 10-, and 15-year OS in the training set (Fig. 8A), and corresponding AUC values of 0.741, 0.712, and 0.745 in the validation set (Fig. 8B), suggesting that the nomogram possesses robust discriminative ability for predicting OS in patients with chondroblastic osteosarcoma.

## Discussion

Chondroblastic osteosarcoma (CBO) is a distinct histological subtype of high grade osteosarcoma, with unique pathogenetic associations with rapid skeletal growth and genetic predisposition^22^. While postoperative chemotherapy has been proven to confer established survival benefits in conventional osteosarcoma, its efficacy in CBO remains controversial due to the limited quantity of relevant evidence and conflicting conclusions among existing studies^12,23–25^. Using data from the Surveillance, Epidemiology, and End Results (SEER) database, this study analyzed 317 patients diagnosed with CBO. Rigorous statistical methods were employed to adjust for treatment selection bias, thereby systematically evaluating the impact of postoperative chemotherapy on overall survival (OS). Furthermore, by integrating CBO relevant factors, we developed and validated a prognostic nomogram capable of predicting 5-year, 10-year, and 15-year OS. This nomogram provides a practical tool for individualized risk assessment in clinical practice.

Our study demonstrated that postoperative chemotherapy was consistently associated with improved OS in patients with CBO (unadjusted Kaplan-Meier analysis: *P* = 0.011; after propensity score matching: *P* = 0.036). This indicates that the observed survival advantage was not attributable to differences in baseline characteristics, but rather to the effect of chemotherapy itself. Subgroup analyses further revealed heterogeneity in the efficacy of chemotherapy. Male patients (HR = 0.48, *P* = 0.01), those with high-grade tumors (HR = 0.54, *P* = 0.015), and individuals from high income groups (≥$90,000; HR = 0.35, *P* = 0.001) derived significant survival benefits from chemotherapy. High income patients exhibited a significantly lower hazard ratio (HR = 0.35), suggesting that socioeconomic factors may modulate the efficacy of chemotherapy. This advantage may stem from their greater accessibility to high volume or specialized treatment centers, where multidisciplinary care and optimized chemotherapy protocols are more readily available^26,27^. Financial stability can also mitigate barriers to treatment adherence, such as the costs of supportive medications and income loss due to treatment-related absenteeism^28^. Related studies have further demonstrated that patients with higher income levels typically access more comprehensive disease information, thereby exhibiting greater initiative in selecting treatment regimens^29^. The study also identified a critical interaction between tumor stage and chemotherapy efficacy (P for interaction = 0.008). Chemotherapy can improve OS in early stage (I–II) patients, but show no significant benefit in advanced stage (III–IV) patients. This finding carries important clinical implications, suggesting that alternative therapeutic strategies—such as targeted therapy or immunotherapy—may be needed for advanced CBO, whereas early stage patients should receive standard chemotherapy. No significant heterogeneity in chemotherapy efficacy was observed across subgroups defined by age, race, marital status, or radiotherapy, indicating that these factors do not modify the fundamental efficacy of chemotherapy.

Multivariate Cox regression analysis identified three independent prognostic factors for CBO, including age, tumor stage and the use of adjuvant radiotherapy. Patients aged 42 years or older had a 2.87-fold higher risk of death compared with younger patients (HR = 2.867, 95% CI: 1.429–5.751, *P* = 0.013), consistent with the widely reported association in the field of osteosarcoma that “older age correlates with poorer prognosis”^30–32^. This association may stem from multiple factors, including higher genomic instability in tumors of elderly patients (e.g., mutations in RB1 and TP53)^33,34^, reduced tolerance to chemotherapy due to comorbidities (e.g., cardiovascular diseases) and lower frequency of monitoring for musculoskeletal symptoms leading to diagnostic delays^35,36^. Using X-tile analysis, we determined 42 years as a novel risk threshold, refining the arbitrary age cutoffs used in prior studies and providing more evidence based guidance for clinical decision-making.

Advanced tumor stage (III–IV) emerged as the strongest adverse prognostic factor (HR = 4.667, 95% CI: 2.073–10.505, *P* = 0.002), consistent with findings from traditional osteosarcoma studies^37,38^. Metastatic CBO, with the lungs being the most common site of metastasis, is associated with extremely poor prognosis, as distant lesions often exhibit intrinsic or acquired resistance to chemotherapy^39–42^. Finally, radiotherapy was identified as a protective factor (HR = 0.449, 95% CI: 0.260–0.775, *P* = 0.016), supporting evidence that adjuvant radiotherapy can improve local control in CBO cases where wide surgical resection is not feasible—such as when the tumor invades neurovascular structures^43,44^.

We developed a CBO specific nomogram incorporating four independent prognostic factors (age, tumor stage, radiotherapy, and marital status) along with chemotherapy status to predict 5-year, 10-year, and 15-year OS. The performance of the nomogram was evaluated using the concordance index (C-index), a metric of predictive accuracy. The training set achieved a C-index of 0.674 (95% CI: 0.619–0.729), and the validation set (*n* = 96) achieved a C-index of 0.691 (95% CI: 0.607–0.775). This level of accuracy was significantly higher than that of mixed subtype osteosarcoma nomograms, highlighting the value of subtype specific modeling. The clinical utility of the nomogram lies in its ability to generate individualized survival probabilities, thereby assisting clinicians in formulating tailored treatment strategies for patients.

Compared with existing studies on osteosarcoma, our research is characterized by its subtype specific focus^14^. Unlike previous nomograms developed for mixed subtype osteosarcoma(such as the model reported by Liu et al. (2023))^18^, our model was specifically designed for CBO and incorporated variables demonstrated to be relevant to this subtype (such as chemotherapy, radiotherapy, and marital status). This specificity addresses the limitations of “one-size-fits-all” models, and the higher C-index provides evidence of significantly improved predictive accuracy. Our nomogram enables the prediction of 5-year, 10-year, and 15-year OS, thereby addressing the need for long-term survival estimation—an area where most existing osteosarcoma models focus predominantly on 5-year outcomes^18,45,46^. This is particularly important for CBO, as patients face a prolonged risk of recurrence after diagnosis, and long term prognostic information is critical for guiding extended care planning, fertility preservation counseling and psychological support. Additionally, we employed propensity score matching to adjust for treatment selection bias—a major limitation in observational studies of rare cancers—thereby rigorously evaluating the efficacy of chemotherapy. This approach effectively isolates the effect of chemotherapy from confounding factors and provides robust evidence that CBO patients benefit from chemotherapy. Most earlier studies on early stage CBO either overlooked selection bias or relied solely on simple regression adjustments, resulting in unreliable conclusions^47^.

Despite its strengths, our study has several inherent limitations as a retrospective analysis based on the SEER database. First, the SEER database does not provide key variables such as surgical margin status (a critical prognostic factor in osteosarcoma), specific chemotherapy regimens (e.g., cisplatin based vs. doxorubicin based), or dose intensity. Notably, surgical margin status is known to influence OS in osteosarcoma and the absence of this variable may introduce residual confounding bias^37^. Second, the SEER database lacks data on local or distant recurrence, limiting our ability to assess the impact of chemotherapy on recurrence free survival. Third, chemotherapy status was recorded in the SEER database as simply ‘yes’ vs. ‘no/unknown.’ This means that some patients categorized as having received ‘no/no documented chemotherapy’ may have actually undergone chemotherapy, although such treatment information was not captured. Lastly, although we performed internal validation using a 7:3 split of the dataset into training and validation sets, external validation using data from independent registries is still required to confirm the generalizability of the nomogram across different patient populations.

## Conclusion​

This study provides definitive evidence that postoperative chemotherapy improves OS in patients with CBO, with particularly significant benefits observed in male patients, those with high grade tumors, and individuals from high income groups. We also identified age, tumor stage, radiotherapy, and marital status as independent adverse prognostic factors. The newly developed CBO specific nomogram offers a practical and accurate tool for individualized risk stratification, filling a longstanding gap in the lack of subtype specific prognostic tools for the diagnosis and management of CBO.

## Supplementary Information

Below is the link to the electronic supplementary material.

Supplementary Material 1

## Data Availability

The datasets generated and analyzed during this study are derived from the ​Surveillance, Epidemiology, and End Results (SEER) Program​ of the National Cancer Institute (https://seer.cancer.gov/). SEER data are publicly available for research purposes upon request through the SEER^*^Stat software (version 9.0.41).
