# Supplementary material for: The effect of postoperative chemotherapy on survival outcomes and a nomogram for predicting overall survival in chondroblastic osteosarcoma
Source: Sci Rep. 2025 Dec 29;15:45055. doi: 10.1038/s41598-025-31032-y (PMC12749957; doi:10.1038/s41598-025-31032-y)
Supplement: Supplementary file 1 — Supplementary Material 1 [file 41598_2025_31032_MOESM1_ESM.docx]

Supplementary Material

Supplementary Fig.1: The flow diagram of matching


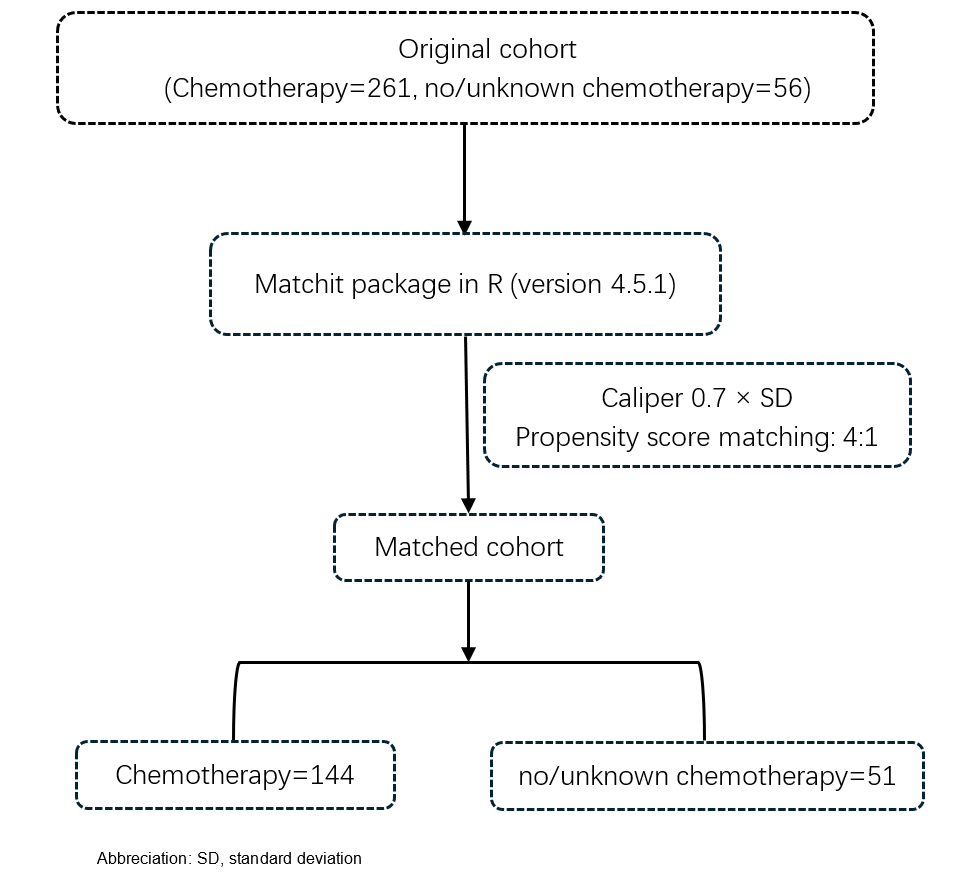


Supplementary Fig.2: The distribution of propensity scores before and after matching

| 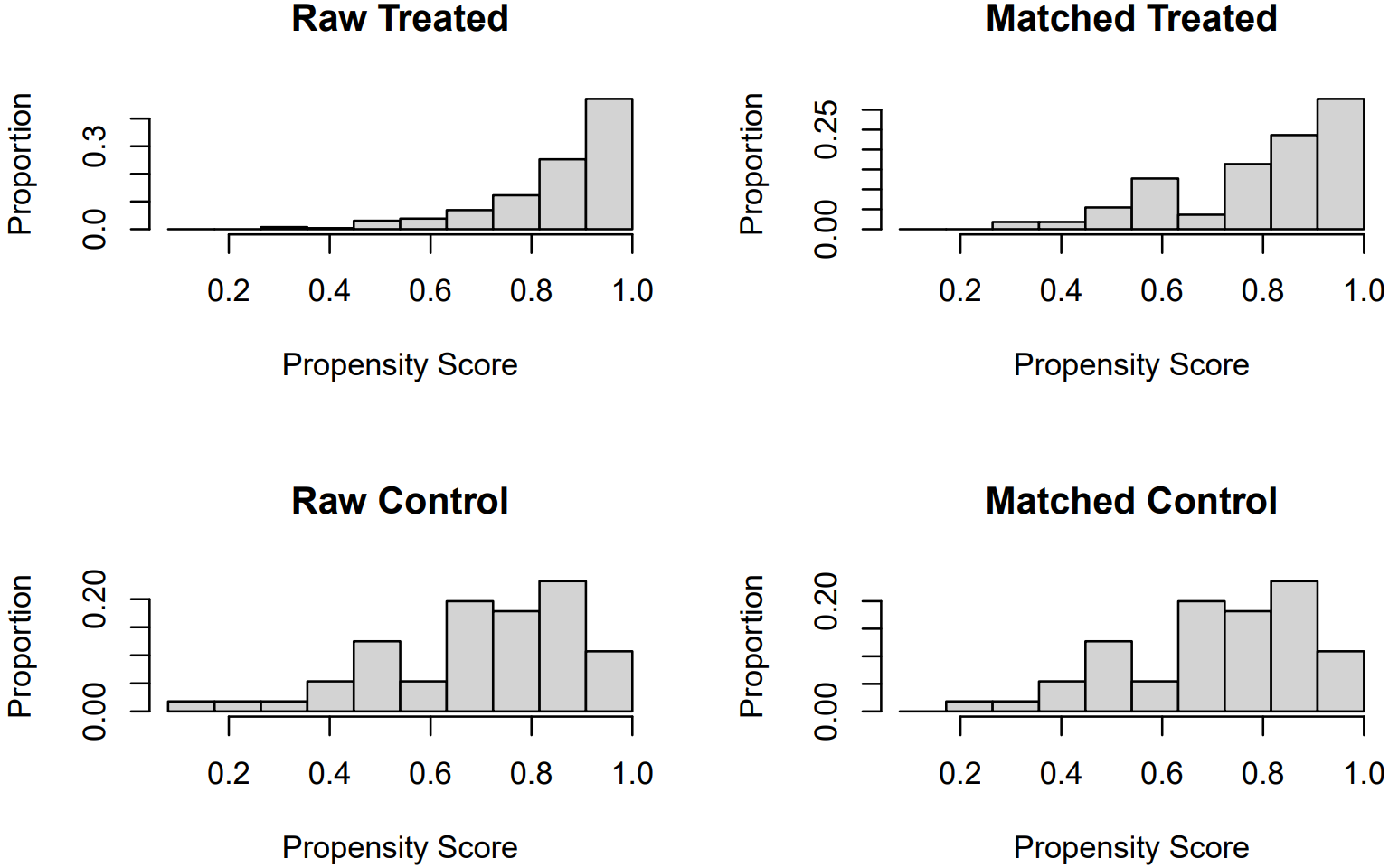 | | | |
| --- | --- | --- | --- |
| Supplementary Table 1: Detailed scores of prognostic factors in the OS nomograms | | | |
| Characteristic | OS nomogram | Characteristic | OS nomogram |
| Stage |  | Radiotherapy |  |
| I-II | 35 | No/Unknown | 4 |
| Unknown | 52 | Yes | 35 |
| III-IV | 100 | Chemotherapy |  |
| Marital status |  | Yes | 23 |
| Unknown | 0 | No/Unknown | 35 |
| Single | 18 | Age |  |
| Married | 19 | <15 | 35 |
| DSW | 35 | 15-42 | 49 |
|  |  | >42 | 80 |
| Abbreciation DSW, divorced&Separated&Widowed | | | |
